# A pilot study of cognitive behavioural therapy integrated with activity pacing for fatigued breast cancer patients undergoing chemotherapy in Ethiopia

**DOI:** 10.3389/fonc.2022.847400

**Published:** 2022-09-21

**Authors:** Mikiyas Amare Getu, Changying Chen, Adamu Addissie, Edom Seife, Panpan Wang, Eva Johanna Kantelhardt

**Affiliations:** ^1^ Department of Oncology, The First Affiliated Hospital of Zhengzhou University, Zhengzhou, China; ^2^ Department of Clinical Nursing, School of Nursing and Health, Zhengzhou University, Zhengzhou, China; ^3^ Global Health Working Group, Martin-Luther-University, Halle (Saale), Germany; ^4^ Institute of Medical Epidemiology, Biostatistics and Informatics, Martin-Luther-University, Halle (Saale), Germany; ^5^ School of Public Health, Department of Preventive Medicine, College of Health Sciences, Addis Ababa University, Addis Ababa, Ethiopia; ^6^ Tikur Anbessa Specialized Hospital, Department of Oncology, College of Health Science, Addis Ababa University, Addis Ababa, Ethiopia; ^7^ Department of Community Health Nursing, School of Nursing and Health, Zhengzhou University, Halle (Saale), Germany; ^8^ Department of Gynecology, Martin-Luther-University, Halle (Saale), Germany

**Keywords:** breast cancer, cognitive behavioural therapy, fatigue, quality of life, depression, pilot study

## Abstract

**Background:**

Fatigue is a common symptom in breast cancer patients, and it is one of the major factors that influence the quality of life (QoL). Cognitive behavioural therapy (CBT) has been recommended to manage cancer-related fatigue. In this study, CBT will be integrated with activity pacing (AP), which can help breast cancer patients achieve a balance between activity and rest. Therefore, this pilot study aimed to investigate the acceptability, feasibility, and efficacy of the CBT-AP intervention.

**Methods:**

A total of 10 fatigued breast cancer patients undergoing chemotherapy were included in the study. The acceptability and feasibility of the study were measured by the patient recruitment rate, attrition rate, intervention fidelity, intervention compliance, and therapist’s and participant’s evaluations of the intervention. The outcomes were measured at baseline and at 6 weeks of intervention.

**Results:**

The pre–post study suggested that CBT-AP was found to be acceptable and feasible for fatigued breast cancer patients undergoing chemotherapy. Among 27 eligible participants, 10 (37.03%) participants accepted our invitation to participate in the study. One participant dropped out from the intervention because of serious illness, and the dropout rate was 10%.

Both the intervention fidelity and intervention compliance were found to be satisfactory.

Fatigue severity [Brief Fatigue Inventory (BFI)] was reduced in 77.77% of participants from baseline to 6 weeks of intervention. The global health status/QoL scale and physical, emotional, and social functioning scales were improved from baseline to 6 weeks of intervention. All symptom scales, except constipation, diarrhea, and financial difficulties, were decreased after the intervention. Depression [Public Health Questionnaire (PHQ)-9] was reduced in 55.55% of participants.

**Conclusion:**

This study suggested that CBT-AP is an acceptable, feasible, and potentially efficacious intervention to reduce fatigue and improve the QoL of breast cancer patients. The efficacy of a CBT-AP programme is going to be investigated in subsequent larger randomized clinical trials.

## Introduction

Breast cancer was the most commonly diagnosed cancer and the leading cause of cancer death among women worldwide in 2020, with an estimated 2.3 million new cases, representing 11.7% of all cancer cases. The incidence and mortality age-standardized rate in Eastern Africa for female breast cancer patients in 2020 were 33% and 17.9%, respectively. Breast cancer is currently the most common type of cancer in Ethiopia with an estimated 16,133 (20.9%) new cases annually and 5-year prevalence of 48.52 per 100,000 (1, 2).

Breast cancer treatment and morbidity related to the disease might present disabling complications on physical functioning, psychological functioning, and behavioural attributes (3) that adversely affect the quality of life (QoL) of cancer patients (4, 5). Patients undergoing chemotherapy most commonly experienced fatigue, pain, and other symptoms (6).

A study conducted in Ethiopia on the QoL of breast cancer patients receiving chemotherapy showed poor QoL among breast cancer patients as compared to other international findings (5). Fatigue, pain, anxiety, and depression are some of the most common problems that occur in breast cancer patients, which negatively affect the QoL of breast cancer patients (7, 8).

Fatigue is a common symptom in breast cancer patients, and it was experienced by 80% of individuals who receive chemotherapy and/or radiotherapy (8–10). A study conducted in Ethiopia among breast cancer patients showed that fatigue is one of the major factors that influence the QoL of breast cancer patients (5).

Breast cancer patients might develop psychological distress and sleep disturbances during the diagnosis, treatment, and posttreatment periods, which negatively influence the QoL (11). A recently conducted systematic review and meta-analysis showed that the global prevalence of depression among breast cancer patients was 32.2%, which is higher in middle-income countries than in developed countries (12). A study conducted in Ethiopia about the prevalence of depression among breast cancer patients reported that one in four (25%) patients had depression. The depression could have a negative impact on adherence to treatment, overall QoL, and survival of the patient (13).

Effective interventions to reduce fatigue and psychological distress during cancer treatment are urgently needed that have potential to improve physical, emotional, and psychological health and overall QoL, as well as to relieve some of the financial burden related to the treatment (14).

Pharmacological interventions such as chemotherapy lead to a wide spectrum of treatment-related disabling complications, such as breast cancer-related lymphedema, pain, bone loss, arthralgia, and fatigue. Therefore, non-pharmacological therapies such as complementary therapies are recommended to reduce disease- and treatment-related symptoms, improve functioning, and improve adherence to treatment and long-term survival of patients (15, 16). To our knowledge, there were no structured non-pharmacologic interventions so far for breast cancer patients and survivors in the center.

Psychosocial interventions specifically designed to treat fatigue were significantly effective in reducing fatigue among cancer patients during cancer treatment (17) and after cancer treatment (18). A meta-analysis study has reported the effectiveness of exercise and psychological interventions in reducing fatigue, and it is significantly better than the available pharmaceutical options. The most effective type of psychological intervention for reducing fatigue was cognitive behavioural therapy (CBT) (19).

The National Comprehensive Cancer Network (NCCN) has recommended CBT to combat cancer-related fatigue (20). CBT is a type of psychological treatment that is effective in managing fatigue (21), anxiety, and depression (22, 23). CBT for insomnia was effective in improving fatigue, anxiety, depression, and QoL (24). In addition, supervised physical exercise was recommended to reduce fatigue and improve the QoL (25).

No previous study has been done to investigate the effect of CBT combined with activity pacing (AP) on breast cancer patients undergoing chemotherapy. Some of the studies were conducted on cancer patients (21), and other studies were conducted on CBT without including AP and did not evaluate cancer-related fatigue as a primary outcome (22, 26). In this study, CBT was integrated with AP (CBT-AP). CBT-AP is based on both the cognitive behavioural theory and energy envelope theory. CBT identifies and replaces dysfunctional thoughts into correct thoughts. The intervention works by acting on the precipitating and perpetuating factors of fatigue (27). In order to act on the precipitating and perpetuating factors, there are different CBT strategies that were used to modify dysfunctional thoughts. These were cognitive restructuring, problem-solving, and coping strategies. The modified thought will bring favourable behaviour and action (28).

AP achieves a balance between activity and rest by energy management. AP enhances physical function and QoL by monitoring energy, fatigue, and activity levels and then modifying daily activities that reduce fatigue and improve the QoL (29). A personalized physical activity schedule has been proven to be effective in combating the adverse reaction of cancer treatment, reducing complications and decreasing mortality due to breast cancer (4).

CBT-AP is hypothesized to be an effective intervention because fatigue is a multidimensional construct (30). Most previous interventional studies conducted on CBT have been carried out on breast cancer survivors (21, 24, 27, 31). However, this study was conducted in patients with early and advanced stages of breast cancer. The earlier treatment of fatigue during chemotherapy is very important because it may assist women not only by decreasing fatigue during cancer treatment but also for treatment adherence. It provides women with evidence-based strategies during their earlier treatment trajectory that they can continue to use after cancer treatment and into survivorship (32, 33). Therefore, it will be a pioneering study that will assess the feasibility and efficacy of CBT-AP among breast cancer patients receiving chemotherapy. The finding of the study will be very helpful to revise the study protocol (33) for the subsequent definitive trial and assess the preliminary efficacy of CBT-AP.

This pilot study aimed to investigate the acceptability, feasibility, and preliminary efficacy of CBT-AP among breast cancer patients undergoing chemotherapy. We hypothesized that the intervention would be feasible and participants would demonstrate greater improvement in fatigue, depression, and QoL at 6 weeks of intervention.

Our research questions were as follows:

1. What is the recruitment and attrition rate?
2. Does the therapist adhere to the intervention protocol?
3. What is the therapist’s and participant’s evaluation of the intervention?
4. Is there any adverse event or serious adverse reactions during the intervention?
5. What is the preliminary efficacy of the intervention?

## Methods

### Design

This is a single-arm pilot study of the CBT-AP Trial (33) that was designed to assess the feasibility, acceptability, and potential efficacy of the intervention. The study was registered at the Pan-African Clinical Trial Registry on 24 August 2020 (PACTR202008881026130). The study has been reported in line with the CONSORT statement (34).

### Participants and study setting

Participants were recruited from Tikur Anbessa Specialized Hospital, Oncology Center and Day-Care Centre, in Addis Ababa, the capital city of Ethiopia from January to June 2021. The sample size was calculated to be 10 by considering 10% of the sample size of the main study.

The inclusion criteria were female breast cancer patients aged 18 years and above who were experiencing severe fatigue (overall rating 7 or more out of 10) for a week or more period on the Fatigue Severity Scale and was non-relapsed in any stage of the disease undergoing chemotherapy.

The exclusion criteria were patients who could not speak Amharic and patients with psychiatric illness or uncontrolled medical illness. Patients were screened by an oncologist for various types of clinically relevant systemic diseases or somatic causes that could result in fatigue (e.g., anaemia, malnutrition, hypothyroidism, and other physical comorbidities). If the fatigue had a somatic cause or was a systemic disease and it was confirmed by the oncologist, the patient did not participate in the study.

The eligibility criteria for the main trial are the same as those for the pilot study.

Consent forms and completed questionnaires were returned directly to the researcher in stamped addressed envelopes (the consent form and the questionnaire were returned in separate envelopes to protect confidentiality). Patients were told that participation was not mandatory.

### Patient recruitment and procedures

Breast cancer patients were invited to participate in the study through brochures and flyers. The names and contact addresses of eligible patients were recorded on the information sheet by the oncologist. When initial eligibility criteria were met, women were contacted by telephone to explain CBT-AP and the study procedures and to confirm their willingness to invest the requested time and effort.

Informed consent was taken from eligible patients who agreed to participate in the study. The participants answered four sets of Amharic version questionnaires as a baseline assessment: the European Organization for Research and Treatment of Cancer Quality of Life Questionnaire Core 30 (EORTC QLQ-C30) (35), European Organization for Research and Treatment of Cancer Quality of Life Questionnaire Breast Cancer Specific Module-45 EORTC QLQ-BR45 (36), Brief Fatigue Inventory (BFI)-9 (37), and PHQ-9 (38). The data collector recorded the sociodemographic and clinical characteristics of the patients from their medical records.

**Figure 1:**
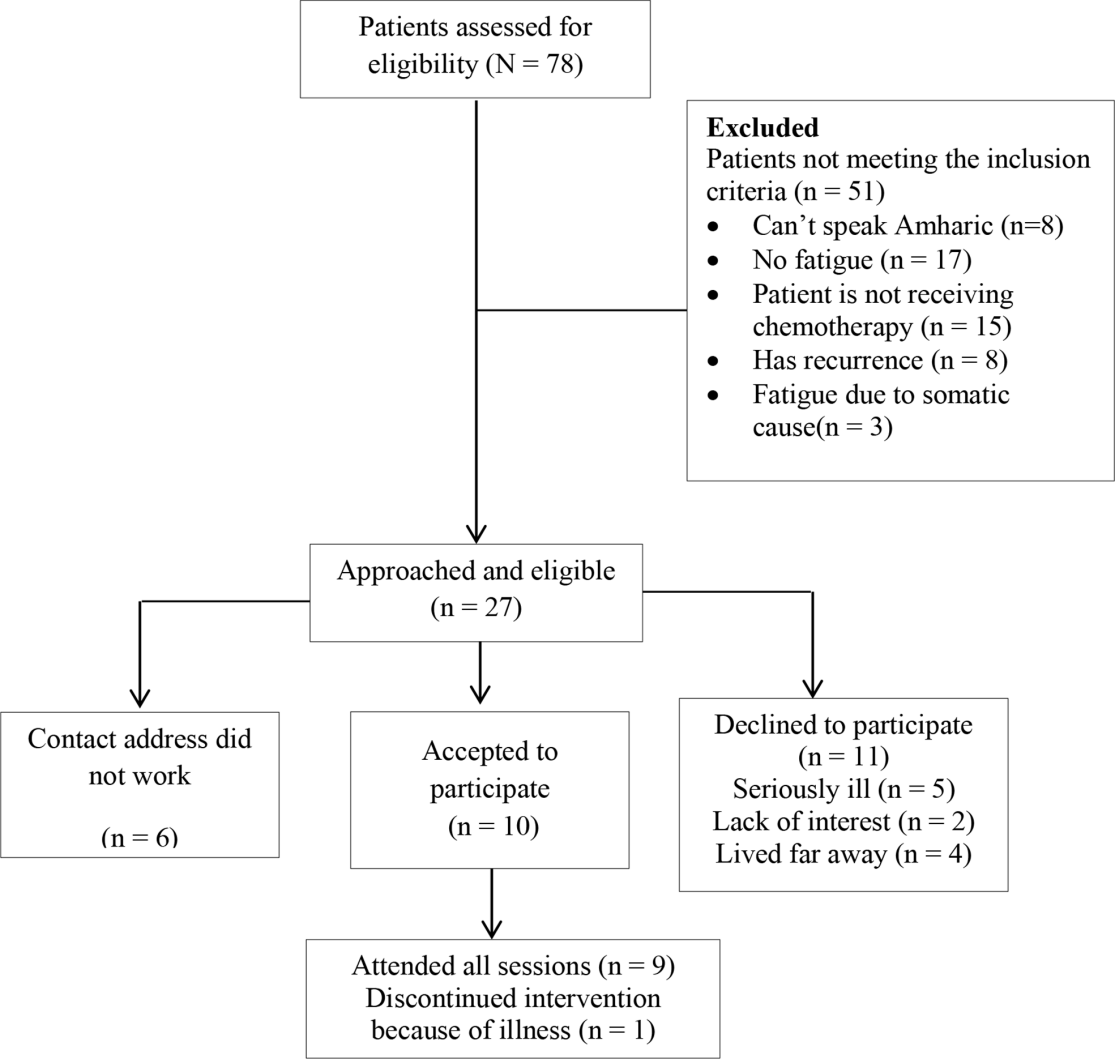
Cognitive behavioural therapy integrated with activity pacing (CBT-AP) patient recruitment CONSORT diagram.

The CONSORT diagram indicating patient flow is depicted in **Figure 1**.

### CBT-AP intervention

The intervention included goal setting, AP, coping strategies, cognitive restructuring, dysfunctional thought management, sleep hygiene, relaxation skills, social support, and realizing goals.

The intervention protocol is developed according to the precipitating and perpetuating factors of fatigue. These include deregulation of activity, dysfunctional cognition, deregulation of the sleep–wake pattern, lack of coping with cancer and its treatment, fear of disease recurrence, and poor social interactions. Furthermore, AP was included in the intervention that is used to encourage patients to avoid exacerbations of fatigue and other symptoms.

The intervention had six sessions: three face-to-face sessions (first, fourth, and sixth sessions) and three telephone sessions (second, third, and fifth sessions). Each session consisted of homework assignments to complete before the next session. The therapists gave instructions on how to do the homework assignments for every session. The manual for the participants and therapists and worksheets for the assignments were prepared and given to the therapists and participants. At the beginning of each session, a revision was done and the participant’s worksheets were evaluated for completion. The intervention was provided by a trained clinical psychologist and oncology nurse.

Therapists completed a 2-day workshop delivered by the developer of CBT-AP.

The average time for the face-to-face sessions was 2 h, while it was 35 min for the telephone sessions. The skill of the interventionist was examined by a standardized checklist.

### Intervention sessions

#### Session 1

During this session, introduction of the therapist and participants and setting of the ground rules for individual and group sessions were made. Introductory information was provided about the disease and its treatment as well as the CBT-AP framework. Progressive muscle relaxation and deep breathing exercises (abdominal breathing) were demonstrated to the participants.

#### Session 2

The participants set SMART—specific, measurable, achievable, and time-bound—goals.

AP was used to balance activity and rest to avoid exacerbation of fatigue.

#### Session 3

Sleep pattern disturbances were managed by maintaining sleep hygiene, avoiding sleeping during the day, and adhering to fixed bedtimes and wake-up times (27, 39).

The participant’s dysfunctional thoughts about breast cancer and its treatment were assessed.

#### Session 4

In this session, participants learnt about cognitive restructuring, which is used to replace unhelpful or dysfunctional thoughts into helpful thoughts. In addition, the therapist explained about coping mechanisms for anxiety, depression, and other diagnosis- and treatment-related problems (40).

#### Session 5

In this session, individual dysfunctional thought management and social support were carried out. The therapist challenged the participant’s dysfunctional thoughts and replaced them with positive/functional thoughts.

#### Session 6

All previous sessions were reviewed, and goals were checked for their achievement. An action plan was prepared to maintain the behaviour.

Details of the intervention are available elsewhere (33).

The content of the CBT-AP outline is shown in **Table 1**.

**Table 1:** Cognitive behavioural therapy integrated with activity pacing (CBT-AP) outline for breast cancer patients undergoing chemotherapy.

Session	Operational procedures	Main themes	Wks.	Delivery	Duration
1	Introduction and setting ground rules Breast cancer, chemotherapy and fatigue Explain CBT framework Relaxation	Introduction of therapist and participants Setting ground rules. Describe breast cancer, chemotherapy, and fatigue. Participants describe experiences of fatigue. Introduce CBT-AP framework and how it manages fatigue and improves the QoL. Introduction to progressive muscle relaxation exercise and deep breathing exercises.	First	Face-to- face	2 h
2	Goal setting Activity pacing	Set realistic goals. Counsel about activity pacing, barriers of activity pacing, managing energy, assess prioritizing daily activities based on available energy and establishing baseline activity.	Second	Telephone	20 min
3	Managing of sleep disturbances Dysfunctional thoughts	Participants identify sleep habits that improve sleep disturbances. Prescribe routine bed time. Consult about sleep hygiene. Identify participant’s dysfunctional thoughts about breast cancer, its treatment, and fatigue.	Third	Telephone	20 min
4	Cognitive restructuring Coping mechanisms	Reformulate dysfunctional cognitions regarding breast cancer, its treatment, fatigue and fear of disease recurrence. Identify reasonable and unreasonable thinking. Coping mechanism to decrease stress, anxiety, depression and treatment side effects.	Fourth	Face-to-face	2 h
5	Individual dysfunctional thought management Improve social support	Counsel on how an individual changes dysfunctional thoughts. Explain how to communicate about fatigue with others. Support system and interpersonal communication. Modify unhelpful cognition about social environment and expectations.	Fifth	Telephone	20 min
6	Realizing goals	Evaluation of the progress with respect to the goal Action plan for maintaining cognitive behavioural change	Sixth	Face-to-face	2 h

### Timing of assessments

Sociodemographic and clinical characteristics, QoL, fatigue, and depression were measured at baseline and after 6 weeks (end of CBT-AP).

### Feasibility outcomes

The feasibility of the outcome was measured by patient recruitment rate, intervention fidelity, intervention compliance, and therapist’s evaluation of the intervention. The acceptability of the intervention was measured by participant’s evaluation of the intervention. The criteria for feasibility of outcomes were considered to be 20% recruitment and attrition rate. A minimum of attending four sessions is the threshold for feasibility to represent sufficient exposure to benefit from the intervention.

### Recruitment rate

Recruitment rate was calculated by dividing the number of enrolled patients by the number of eligible patients.

### Intervention fidelity

The feasibility outcomes were evaluated based on intervention fidelity and compliance with the programme. A standardized checklist was used to assess intervention fidelity and compliance.

Acceptability was measured based on the participants’ evaluations of the intervention. The intervention fidelity of the therapy was monitored by an assessor. All face-to-face sessions and telephone sessions were recorded after getting consent from participants. The assessor rated the intervention fidelity using a standardized fidelity checklist. The intervention fidelity was scored on a scale from 0 (poor) to 5 (excellent) based on the following criteria: 1) The content of the session was covered; 2) Group/individual discussion points were raised and discussed very well; 3) An explanation was given about the homework, and the therapist strongly recommended them to read the module and do their homework; 4) The time taken was within the proposed range; 5) The participants were given the opportunity to explain their ideas; 6) The participants were interested and actively participating in the session; 7) The participants did their homework; and 8) The participants attended sessions on time during the face-to-face/telephone sessions.

The checker was a registered psychiatric nurse with a clinical experience of 7 years and trained on the CBT-AP intervention.

In order to increase the intervention fidelity, the intervention provider was provided with a therapist intervention manual that included all materials necessary to effectively deliver the intervention.

### Participant’s evaluation of the therapy

Upon completion of the programme, the participants were asked to complete a short evaluation that included statements about their satisfaction with different elements of the programme as assessed by a 4-point Likert scale: “not at all”, “a little”, “quite a bit”, and “very much”. In total, 14 statements were presented.

### Intervention outcomes

#### Primary outcome measure

##### Fatigue

Fatigue was measured by the Brief Fatigue Inventory Amharic version (BFI-Amh) (37). The BFI consists of nine items asking patients whether they felt unusually fatigued in the last week. The BFI-Amh showed good acceptability, internal reliability (Cronbach’s α = 0.97), construct, and concurrent validity (37).

#### Secondary outcome measures

##### Quality of life

###### Eortc Qlq-C30

It is a reliable and valid measure of the QoL of cancer patients. It consists of 30 items arranged with five functional scales, three symptom scales, and a global health and QoL scale. According to scoring procedures, the QLQ-C30 was transformed to scores ranging from 0 to 100. Higher scores represent a better level of functioning on the functional and single-item scales. A higher score of symptom scales represents a higher level of symptoms for the symptom scales (35). The score of each item ranged from 1 (not at all) to 4 (very much).

###### Eortc Qlq-BR45

The updated version of the EORTC QLQ-BR23 incorporated an additional 22 items that contain a target symptom scale and satisfaction scale (36). The EORTC QLQ-BR45 had good internal reliability (α = 0.80), test–retest reliability (α = 0.77), and validity. The score of each item ranged from 1 (not at all) to 4 (very much).

##### Depression

The PHQ-9 is a short tool used to assess depression. The Amharic version of the PHQ-9 demonstrated good internal reliability (α = 0.81), test–retest reliability (α = 0.92), and validity results (38). The score of each item ranged from 0 (not at all) to 3 (nearly every day). The total score ranges from 0 to 27 (41).

This pilot study is not blinded. So, the analysis was done by the principal investigator who is a PhD student in Nursing. However, the randomized controlled trial (RCT) that we have conducted after the pilot study was blinded for the outcome assessors and statistician. Therefore, an independent statistician who was blind to the treatment allocation did the data analysis.

### Statistical analysis

Statistical analysis was principally descriptive, and the frequency, mean, and standard deviation (SD) were calculated. We assessed the acceptability of CBT-AP by the therapist’s and participant’s evaluations of the intervention and number of CBT-AP sessions attended.

The feasibility of the study was assessed in terms of recruitment rate into the study (number agreeing to participate out of those approached), attrition rate, and adherence to and compliance with the intervention. The preliminary efficacy of the therapy was examined by differences before and after the intervention, and data from all participants (N = 9) were analysed using descriptive statistics such as frequency, mean, and SD.

### Ethics approval

All procedures performed in studies involving human participants were in accordance with the ethical standards of the institutional and/or national research committee and with the 1964 Helsinki declaration and its later amendments or comparable ethical standards. The study was approved by the ethics committees of the Zhengzhou University IRB (number: ZZURIB 2020-10; Date: 18 June 2020) and Addis Ababa University, College of Health Science teaching hospital (number: 101/20/Onco; Date: 28 October 2020). The patients/participants provided their written informed consent to participate in this study.

## Results

### Demographic and clinical characteristics

In total, 10 women participated in the study. Nine (90%) patients completed all sessions. Participants had a mean age of 38.3 years, SD = 7.23.

The majority (80%) of participants had undergone surgery, 20% had hormonal therapy, and 50% had received surgery and chemotherapy. Half of the participants (50%) had secondary education. Half of the participants were housewives. Majority (90%) of the participants were living in an urban area.

Most of the participants (50%) had a stage III tumour, and the histological classification of the tumour for all participants was ductal carcinoma (**Table 2**).

**Table 2:** Baseline sociodemographic and clinical characteristics of breast cancer patients undergoing chemotherapy.

Variables	Frequency	Percentage
Age Mean (SD)	38.3 (7.2)	
**Educational status**		
No formal education	2	20
Primary education	1	10
Secondary education	5	50
Above secondary education	2	20
**Occupational status**		
Housewife	5	50
Government employed	1	10
Merchant	1	10
Daily labourer	3	30
**Religion**		
Orthodox Christianity	8	80
Muslim	1	10
Protestant	1	10
**Residence**		
Urban	9	90
Rural	1	10
**Marital status**		
Single	3	30
Married	3	30
Divorced	2	20
Widowed	2	20
**Stage of tumour**		
Stage I	1	10
Stage II	2	20
Stage III	5	50
Stage IV	2	20
**Cancer treatment** Chemotherapy Surgery Radiotherapy Hormonal therapy Surgery + Hormonal therapy + Chemotherapy Surgery + Chemotherapy **Monthly income (ETB)**	10 8 0 2 2 5	100 80 0 20 20 50
≤2,000	7	70
2,001–3,000	1	10
>3,000	2	20
**ECOG-PS**		
ECOG I	7	70
ECOG II	2	20
ECOG III	1	10
ECOG IV	0	0
**Chemotherapy cycle**		
1–4	6	60
5–8	4	40
**Comorbidity**		
Yes	4	40
No	6	60
**Time since diagnosis (month)**		
1–6	6	60
7–12	4	40

SD, standard deviation; ECOG-PS, Eastern Cooperative Oncology Group Performance Status; ETB, Ethiopian birr.

### Participant’s evaluation of the therapy

All participants (100%) reported having received sufficient information about breast cancer, treatment approach, and benefits of the therapy. Most women reported that they had sufficient progress after the intervention (88.9%) and had more control over their symptoms after the therapy (66.7%). Most of the qualities of the therapist, such as expertise, trustworthiness, punctuality, and respectfulness, were evaluated positively (100%). Most women were satisfied with the module (66.7%), thought their treatment was relevant and the correct approach (100%), and telephone session was easily delivered and understandable (100%). Moreover, the participants advised others patients to attend this therapy (100%) (**Table 3**).

**Table 3:** Participant’s evaluation of the intervention among breast cancer patients.

Information	Not at all	A little	Quite a bit	Very much
I received sufficient information about breast cancer, types of treatment, treatment side effects, and management.	_	_	_	100
I have received sufficient information about the benefits of the therapy.	_	_	_	100
**Experienced effect**				
I had sufficient progress after the intervention.	_	_	11.1	88.9
Because of the intervention, I had control over my symptoms.	_	11.1	22.2	66.7
**Therapist**				
The therapists had sufficient expertise and delivered all sessions successfully.	_	_	_	100
I trusted the therapists.	_	_	_	100
The therapists were respectful.	_	_	_	100
The therapists were punctual.	_	_	_	100
The therapists were interested in me and my opinion.	_	_	22.2	77.8
The therapists provided an immediate response to emergency situations.	_	_	_	100
The therapists explained the content of the session at the beginning of each session.	_	_	11.1	88.9
**Other**				
I am satisfied with the module prepared for the participants.	_	_	33.3	66.7
The telephone session was easily delivered and understandable.	_	_	_	100
The treatment was relevant and the correct approach for my symptoms.	_	_	_	100
I would advise others to follow this therapy.	_	_	_	100

### Therapist’s evaluation of the therapy

The therapists were generally satisfied with the content of the therapy but suggested to revise the Amharic translation of a few vague words in the module and to increase the telephone session duration from 20 min to 30–35 min. Session 1 took 2 h and 40 min, which is longer than the proposed time. They have also suggested including “brainstorming” questions at the beginning of every module in which the participants could actively participate and give reflection on the concepts of the session. Appendices “C” and “H” of the worksheets were suggested for revision to be more easily understood by the participants.

### Feasibility of the intervention

#### Recruitment

A total of 78 patients were assessed for eligibility. Of those assessed for eligibility, 51 patients did not meet the inclusion criteria, leaving 27 patients eligible. Among the 27 eligible participants, 10 (37.03%) participants accepted our invitation to participate in the study, and the recruitment rate was 37.03%. The remaining 17 patients did not participate in the study because six of them had contact addresses that were unreachable and 11 of them declined to participate in the study. Reasons for declining participation included being seriously ill (n = 5), lack of interest (n = 2), and living too far away (n = 4).

### Intervention fidelity

The intervention fidelity and intervention compliance were satisfactory. According to the standardized checklist, all of the session content and objectives were well addressed (100%). The group/individual discussion points were discussed very well, and an explanation was given about the homework (100%).

The participant’s punctuality (73.3%) and therapy session were within the proposed time range (66.7%). Completion of homework by participants was not applicable during sessions 1 and 6. In the other sessions, the completion rate of homework by participants was 50%.

### Intervention compliance

Intervention compliance was defined as the number of sessions completed and time spent in each session of the therapy. The study participants were expected to attend six sessions. Among 10 patients, seven patients attended all (100%) of the sessions. Two patients attended four (66.7%) sessions and missed another two sessions because of illness. One participant dropped out from the intervention because of serious illness, and the dropout rate was 10%. There were 54 sessions in total for these nine patients. Upon completion of the study, 50 (93%) out of 54 expected sessions had been completed by nine participants. A missed session was replaced by another telephone session within 5 working days. Therefore, the overall compliance rate by session was 93%.

### Outcome measures

#### Primary outcome measure

##### Fatigue

**Figure 2:**
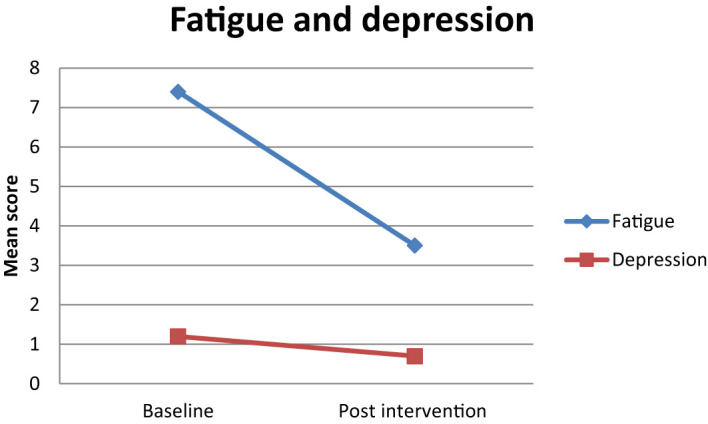
Estimated mean trajectories of fatigue and depression among breast cancer patients.

Fatigue severity (BFI-9) decreased in 77.77% of subjects from a mean of 7.40 ( ± 2.21) at baseline to 3.50 ( ± 1.94) at 6 weeks of intervention (P ≤ 0.05) (**Figure 2**; **Table 4**).

### Secondary outcome measures

#### Quality of life

##### Eortc Qlq-C30

**Figure 3:**
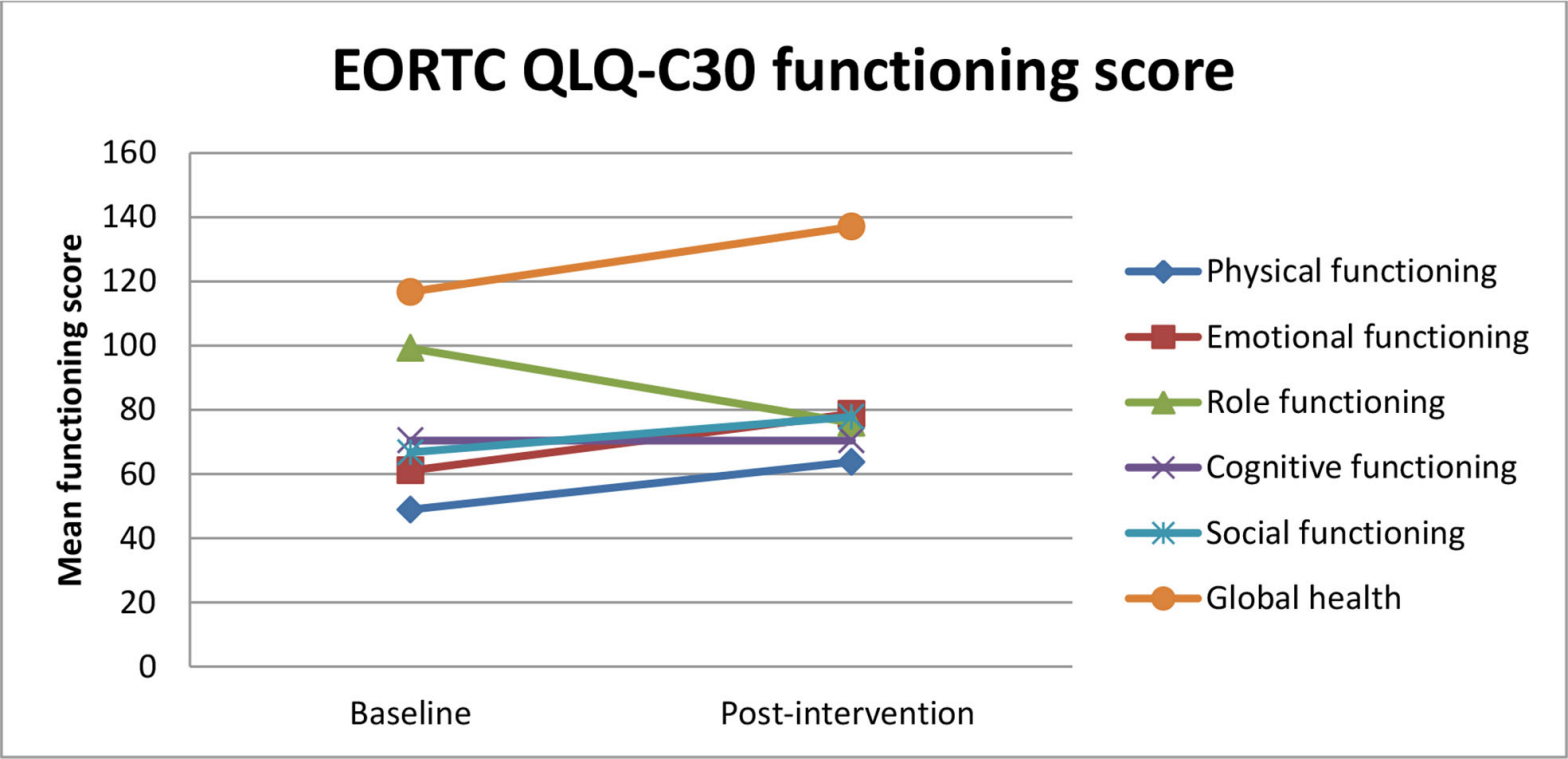
Estimated mean trajectories of quality of life functioning scores among breast cancer patients.

The global health QoL scale and physical, emotional, and social functioning scales were improved from baseline to 6 weeks of intervention (**Figure 3**).

All symptom scales, except constipation, diarrhea, and financial difficulties, were decreased after the intervention (**Figure 2**; **Table 4**).

The effect of CBT-AP on EORTC QLQ-C30 functioning and symptom score is shown in **Table 4**.

**Table 4:** The effect of Cognitive behavioural therapy integrated with activity pacing (CBT-AP) on fatigue, depression, and quality of life of breast cancer patients undergoing chemotherapy.

EORTC QLQ-C30Domain/Symptom	Baseline		Posttest	
	Mean	SD	Mean	SD
Global	116.7	61.8	137.0	41.5
Physical	48.9	27.5	63.7	26.5
Role	99.3	17.7	75.9	29.0
Emotional	61.1	27.6	78.7	27.9
Cognitive	70.4	27.4	70.4	18.6
Social	66.7	39.1	77.8	25.0
Fatigue	70.4	27.7	39.5	28.4
Nausea and vomiting	59.3	45	42.6	37.4
Pain	72.2	38.2	35.2	33.8
Dyspnea	37.0	48.43	18.5	29.4
Insomnia	48.1	37.7	29.6	35.1
Appetite loss	66.7	44.1	33.3	28.9
Constipation	22.2	28.9	25.9	36.4
Diarrhea	11.1	23.6	11.1	23.6
Financial difficulties	44.4	47.1	59.2	46.5
**EORTC QLQ-BR45** **Domain/Symptom**				
Body image	70.4	22.5	87	27.4
Sexual functioning	98.1	5.5	98.1	5.5
Sexual enjoyment	100	0.0	100	0.00
Future perspective	40.7	40	74.1	43.4
Systematic therapy side effect	52.4	22.3	39.7	16
Breast symptom	29.6	24.3	25.9	18.4
Arm symptom	37	28.3	22.2	19.2
Upset by hair loss	50	35.6	81.5	37.7
Breast satisfaction scale	51.8	46.7	75.9	40.1
Endocrine therapy scale	31.5	15.6	22.2	11.9
Endocrine sexual scale	-29.6	11.1	-29.6	11.1
Skin mucosis scale	21.6	15.8	17.3	13.7
**Fatigue (BFI-9)** **Depression (PHQ-9)**	7.40 1.20	2.21 0.76	3.50 0.70	1.94 0.51

EORTC QLQ-C30, European Organization for Research and Treatment of Cancer Quality of Life Questionnaire-Core30; EORTC QLQ-BR45, European Organisation for Research and Treatment of Cancer Quality of Life Questionnaire- Breast Cancer Specific Questionnaire45; BFI-9, Brief Fatigue Inventory-9; PHQ-9, Patient Health Questionnaire- 9; SD, Standard deviation.

##### Eortc Qlq Br45

Body image, future perspective, and breast satisfaction functioning scales were improved after the intervention. All symptom scales except upset by hair loss and endocrine sexual scales were decreased from baseline to 6 weeks of intervention.

**Figure 4:**
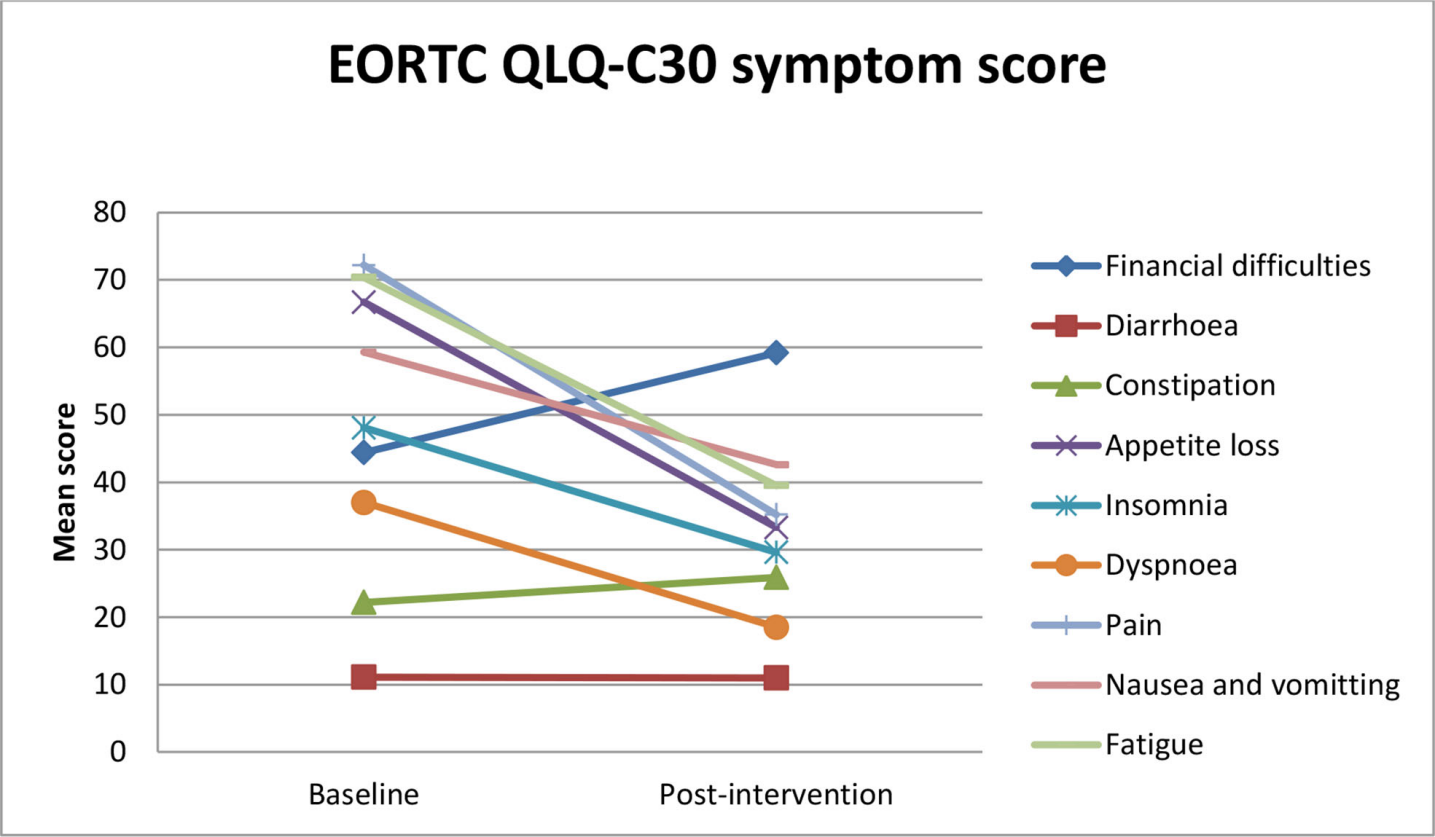
Estimated mean trajectories of quality of life symptom scores among breast cancer patients.

A reduction in the symptom scale (fatigue, pain, dyspnea, insomnia, and appetite loss) was reported (**Figure 4**).

#### Depression

Depression measured by PHQ-9 was reduced in 55.55% of participants from a total mean score of 1.20 ( ± 0.76) at baseline to 0.70 ( ± 0.51) after intervention (**Figure 2**; **Table 4**).

#### Adverse events

There were no adverse events or serious harm that occurred during the intervention.

## Discussion

### Feasibility of intervention

The cancer tumor-related factors and its treatment lead to disabling complications such as fatigue and pain that will directly affect the QoL.

This is the first study conducted to evaluate the feasibility of CBT-AP and to provide preliminary data about its effect on fatigue, depression, and QoL among breast cancer patients. In this study, CBT-AP was found to be a feasible and potentially efficacious intervention for breast cancer patients undergoing chemotherapy. Another study conducted on cancer patients had shown that self-help workbook intervention was feasible in cancer patients receiving chemotherapy, although the effect of the intervention was limited (42). The discrepancy between the effects of intervention might be because the intervention was guided by a trained therapist in this study.

The intervention fidelity and the intervention compliance were satisfactory according to the checklist. Participants’ attendance to each session was good, with seven participants among 10 who attended all of the sessions (100%). Two patients attended four (66.7%) sessions. We considered attendance at a minimum of four sessions to represent sufficient exposure to benefit from the intervention.

However, the percentage of participants who completed their homework/worksheet (a type of task used to exercise CBT-AP on a daily basis) at each session was relatively low (50%). The main reasons mentioned by the patients were being “unable to comprehend the instructions for the homework/worksheets and some vague words” and illnesses during the therapy. Revision was recommended on the worksheet instructions and the replacement of a few words for easy understanding in the main trial. The time allowed for session 1 (face-to-face) and all of the telephone sessions was inadequate. Therefore, the telephone session was suggested to be delivered for 30–35 min, and session 1 was suggested to be delivered in two sessions.

The participant’s punctuality for the face-to-face session was not satisfactory. This might be due to participants residing far from the centre where the therapy was delivered and lack of adequate transportation. Therefore, the participants should be informed over the telephone 1 day earlier than the face-to-face session about the time schedule and to get well prepared for early transportation access.

Some patients declined to participate in the study after recruitment due to lack of interest, living too far away, longer therapy sessions, and unable to reach their contact addresses. Future efforts should focus on explaining about the benefits of participating in the study before requesting informed consent, taking additional contact address, and scheduling interventions and evaluations for the same day as chemotherapy sessions to optimize patient participation and to avoid declining to participate. Other strategies should be suggested to increase the number of participants and prevent dropout, such as regularly calling patients weekly to strengthen their participation and minimize the dropout rate (43).

### Efficacy of CBT-AP

Our pilot study showed that breast cancer patients undergoing chemotherapy who received our novel CBT-AP intervention demonstrated decreased fatigue from baseline (mean = 7.40, SD ± 2.21) to 6 weeks of intervention (mean = 3.50, SD ± 1.94) as measured by BFI-9, and EORTC QLQ-C30 fatigue scale showed decreased fatigue (from a mean = 70.4, SD ± 27.7, to a mean = 39.5, SD ± 28.4). These results demonstrate the preliminary efficacy of our novel CBT-AP as an active fatigue intervention. This is consistent with previous studies done on the effect of CBT among cancer patients (39, 44) and cancer survivors (21). Similarly, another study showed that rehabilitative physical exercise was found to be safe, feasible, and effective to reduce fatigue (45). This can be explained by increased functioning scales, and decreased symptom scales contributed to the reduction of fatigue. In this study, the effect of CBT-AP on the QoL might be attributed to the reduction of fatigue. These results highlighted that the effect of CBT-AP in reducing fatigue had contributed to improve the QoL of breast cancer patients. Contrary to this study, a systematic review and meta-analysis of physical therapies showed no effect on the reduction of fatigue. This might be due to heterogeneity of the included studies (46).

Invernizzi et al. (45) revealed the preliminary efficacy of rehabilitative activity in improving EORTC QLQ-C30 functional score and global health status score and reduction of EORTC QLQ-C30 symptom scales. In this study, physical function, emotional function, social function, and global health status/QoL subscales were improved after intervention. Similarly, another study showed improvement in the social and emotional functioning scale of EORTC QLQ-C30 (23). Zhu XY, Li Z, Chen C, et al. (46) found that a meta-analysis showed that physical therapies improved the overall QoL of breast cancer patients. Fatigue, pain, dyspnea, insomnia, and appetite loss were decreased from baseline to post intervention, which is in agreement with previous studies conducted on the effects of psycho-education on breast cancer patients (23). Moreover, CBT had shown improvements on fatigue from baseline to 15 months of therapy (47).

According to this study, an increase in functioning scales and decrease in symptom scales of EORTC QLQ-C30 and EORTC QLQ-BR45 might contribute to reducing fatigue among breast cancer patients.

The findings of this study showed that constipation, diarrhea, and financial difficulties were increased from baseline to 6 weeks of intervention. This can be explained by the fact that CBT-AP is not a valid intervention for constipation and diarrhea. Similarly, there is no financial-related content in the intervention sessions; thus, it may not be valid for financial difficulties too. Previous studies have shown the efficacy of CBT for depression in breast cancer patients (48, 49). In this study, there was a minimal decrease in the depression scale. This might be due to the small sample size in this study. The forthcoming trial will consider implementing different ways to improve the patient recruitment rate, participant retention, and compliance with the intervention. The efficacy of a CBT-AP programme is going to be investigated in larger RCTs.

CBT-AP is safe and can be feasibly implemented within the standard of care for the future larger trial. CBT-AP shall be integrated to routine cancer care based on the findings of the main trial.

### Strengths and limitations

While this study produced intriguing results on the preliminary efficacy of the new intervention, there are several important limitations to note. The limitation of this pilot study was the small sample size, which could not allow us to conduct statistical analysis of the differences between the baseline and 6 weeks of intervention for fatigue, depression, and QoL variables and decreased the generalizability of the findings. Other previous pilot studies had also faced small sample sizes (50, 51). The small sample size in this study was due to the nature of the study (a pilot) and lack of eligible participants.

We would emphasize that these results need to be interpreted with caution because of the design of pre–post pilot study with a small sample size and the limited length of observation time. However, the findings of the study were sufficiently promising to justify conducting an RCT, which we are currently doing.

Comorbidities and their medication that may affect the outcome were not included into the questionnaire. Since those are rare in the population, we expect little bias.

The recruitment rate was found to be low. Therefore, modification of the recruitment method is recommended in the subsequent trial. Tracking system should be established for each of the individuals to improve the recruitment rate and recruiters to follow each individual through the entire process. It is also recommended to explain the study in more detail, i.e., benefits and risks of participation to improve their interest to participate, answer patient’s questions, tracking with an alternative phone number to solve unreachable address, to cover the transportation cost for those who lived far away, and increase the recruitment period to achieve an adequate sample size.

## Conclusion

The findings of this study indicated relevant improvement of fatigue from baseline to 6 weeks of intervention. CBT-AP was found to be a safe, feasible, and potentially efficacious intervention to reduce fatigue and improve the QoL of breast cancer patients. The results of our prospective randomized clinical trial will provide more definitive information regarding the efficacy of the intervention.

## Data availability statement

The original contributions presented in the study are included in the article. Further inquiries can be directed to the corresponding authors.

## Ethics statement

All procedures performed in studies involving human participants were in accordance with the ethical standards of the institutional and/or national research committee and with the 1964 Helsinki declaration and its later amendments or comparable ethical standards. The study was approved by the ethics committees of the Zhengzhou University IRB (number: ZZURIB 2020-10; Date: 18 June 2020) and Addis Ababa University, College of Health Science teaching hospital (number: 101/20/Onco; Date: 28 October 2020). The patients/participants provided their written informed consent to participate in this study.

## Author contributions

MG is responsible for the conception and design of the study. CC, EK, ES, and AA have been supervising the overall activities and provided valuable comments. MG, CC, EK and WP have been involved in data analysis, and interpretation. All authors contributed to the article and approved the submitted version.

## Funding

This work was supported by Else-Kroener-Foundation through Martin-Luther-University, Halle-Wittenberg, Germany [grant No. 2018_HA31SP]. The funding body did not have any role in the study design, data collection, analysis and interpretation of data, and in writing the manuscript. The open-access publication fee is received from Zhengzhou University, School of Nursing and Health.

## Acknowledgments

We express our gratitude to the patients who participated in this study. We are also grateful to Addis Ababa University, College of Health Science for arranging a room to deliver the therapy to the patients.

## Conflict of interest

The authors declare that the research was conducted in the absence of any commercial or financial relationships that could be construed as a potential conflict of interest.

## Publisher’s note

All claims expressed in this article are solely those of the authors and do not necessarily represent those of their affiliated organizations, or those of the publisher, the editors and the reviewers. Any product that may be evaluated in this article, or claim that may be made by its manufacturer, is not guaranteed or endorsed by the publisher.

## Abbreviations

AP, activity pacing; BFI, Brief Fatigue Inventory; CBT, cognitive behavioural therapy; EORTC QLQ-C30, European Organisation for Research and Treatment of Cancer-Quality of Life Questionnaire-Core 30; TASH, Tikur Anbessa Specialized Hospital; NCCN, National Comprehensive Cancer Network; PHQ, Public Health Questionnaire; QoL, quality of life; SD, standard deviation.
